# Modulating Ubiquinone Biosynthesis Rescues Mitochondrial Dysfunction by Genetic Interaction in a Phosphatidylethanolamine (PE)-Deficient Disease Model

**DOI:** 10.3390/antiox15091212

**Published:** 2026-09-20

**Authors:** Lavanya Vadupu, Vijay Aditya Mavuduru, Writoban Basu Ball

**Affiliations:** Mitochondrial Biogenesis Lab, Department of Biological Sciences, School of Engineering and Sciences, SRM University-AP, Amaravati 522240, Andhra Pradesh, India; lavanya_vadupu@srmap.edu.in (L.V.); vijayaditya_m@srmap.edu.in (V.A.M.)

**Keywords:** *Caenorhabditis elegans*, phosphatidylethanolamine, *psd-1*, *clk-1*, RNA interference, retrograde signaling, reactive oxygen species, mitochondrial rescue, DAF-16/FOXO

## Abstract

Mitochondrial dysfunctions are often associated with cellular aging as well as metabolic disorders. Therapeutic strategies for rewiring mitochondrial stress signaling in a disease context could be beneficial. New evidence indicates that mitochondrial membrane lipids actively regulate mitochondrial signaling pathways that influence cellular health, stress responses, and longevity. Phosphatidylethanolamine (PE), the non-bilayer-forming phospholipid, plays major roles in mitochondrial morphology and ETC function. The biosynthesis of PE takes place within the inner mitochondrial membrane and is catalyzed by the enzyme phosphatidylserine decarboxylase-1 (PSD-1), which converts phosphatidylserine (PS) to PE. While the biochemical role of PSD-1 in PE biosynthesis is well established, how its dysfunction translates into mitochondrial pathology remains poorly understood. In this work, we describe the physiological implications of PSD-1 function and validate a novel disease model for human *PISD* (the ortholog of *C*. *elegans psd-1*) by undertaking targeted gene knockdown using RNA interference (RNAi) in *Caenorhabditis elegans*. We show that *psd-1* deficiency causes several physiological defects in *C. elegans*, demonstrating that PE biosynthesis via PSD-1 is critical for mitochondrial activity and organismal development (mitochondrial PE levels were reduced by 69.51% in *psd-1* knockdown worms compared to controls). These findings establish *psd-1* knockdown worms as a reliable model for studying human *PISD*-related disease. Surprisingly, co-knockdown of *psd-1* along with the ETC component *clk-1* rescued physiological defects such as restoring the lifespan of from 13.48 ± 0.51 days *psd-1* RNAi worms to 17.36 ± 0.44 days in *psd-1*; *clk-1* double-knockdown worms and normalizing oxygen consumption rate, suggesting that ETC-mediated retrograde signaling, rather than phospholipid depletion alone, is the primary driver of the observed pathology.

## 1. Introduction

Although energy production is the traditional function of mitochondria, it has been recently acknowledged as a signaling organelle influencing the decisions of cellular and organismal growth, development, stress response, and longevity [1]. Their signaling capacity is strongly connected to their structural integrity. Mitochondria continuously monitor cellular homeostasis by producing biochemical signals such as ROS, calcium fluxes and other byproducts [2]. These molecules are the messengers of retrograde signaling which transfers information to the nucleus, thus inducing protective gene expression [3]. However, when mitochondrial lipid homeostasis is compromised, excessive ROS are generated, which damage intracellular signaling networks and override these defense pathways [4], ultimately leading to age-related and neurodegenerative diseases [5].

The mitochondrial inner membrane (IMM) is a dynamic foundation with its unique cristae architecture, which is essential for oxidative phosphorylation and signaling complex assembly [6]. The balance between bilayer-forming (PS, PC) and non-bilayer phospholipids (PE, CL) is crucial for preserving cristae structure and function [7]. Among these, PE and CL promote the stability and assembly of electron transport chain (ETC) complexes and support the mitochondrial cristae network [8,9]. Cardiolipin (CL) is a signature phospholipid of the IMM, while PE is the major non-bilayer-forming phospholipid. Both are synthesized directly within the IMM, and disruptions in their metabolism are frequently implicated in severe human disorders. Structural and functional studies have identified that PE binding is required for cytochrome bc1 complex (Complex III) activity in yeast and human mitochondria, and a resolved PE lipid-binding site has also been reported in respiratory Complex I [8,10], indicating that PE is not only a component of the membrane but may function as a direct anchor for ETC assembly.

Focusing on its clinical importance in humans, *PISD*, the human ortholog of *C. elegans psd-1*, is a mitochondrial disease gene associated with skeletal dysplasia and cataracts [11]. A recent case study also revealed that mutations in this human PISD gene cause Liberfarb syndrome, an extremely rare multisystem genetic disorder that affects ear, eye, brain and bone development [12]. Also, deletion of *psd1* (*PISD* in mammals and *PSD1* in yeast) showed decreased levels of PE, reduced cellular growth, impaired respiration and disrupted mitochondrial morphology highlighting the importance of PE in mitochondrial and overall cell health [13].

Because PISD-related pathology remains poorly understood and no genetic modifiers of phospholipid deficiency phenotypes have been identified, we considered whether other mitochondrial pathways might functionally interact with PSD-1 loss. One such candidate is ubiquinone (CoQ) biosynthesis, because a link between phospholipid metabolism genes and CoQ regulation has been noted in a yeast genetic screen [14], though not as a direct, tested genetic interaction. To our knowledge, this is the first study to demonstrate a genetic interaction between a phospholipid biosynthesis gene and an ETC/CoQ pathway gene in a whole-animal model. Depletion of PE methyltransferase *CHO2* in yeast upregulated the CoQ levels by increasing the SAM-mediated methylation steps in CoQ biosynthesis [14]. This observation indicates a conserved compensatory response to maintain and restore redox balance and mitochondrial respiratory capacity. These clinical and preclinical studies highlight the significance of mitochondrial lipids as active regulators of mitochondrial functions as well as reveal possible ways to target lipid-mediated mechanisms [15]. Even though the biochemical role of PSD-1 in PE biosynthesis and mechanisms regulating electron transport chain has been well documented, PISD disease pathology remains poorly understood at the molecular level. This is because mammalian homozygous knockouts have been embryonically lethal making it difficult to study post-natal development and adult disease phenotypes [16]. While yeast and cultured mammalian cells have provided valuable insight into these processes, these models cannot fully reveal the tissue-level metabolic interactions at the organismal scale. Hence, the direct genetic link between mitochondrial phospholipid biosynthesis and ubiquinone metabolism remains unexplored in an intact animal model.

Given the direct involvement of PISD in mitochondrial diseases in human, we have addressed a few important questions using a nematode model of mitochondrial PE depletion in *Caenorhabditis elegans*. Our study investigates the impact of *psd-1* knockdown on physiological functions, longevity and mitochondria-regulated disease susceptibility. Additionally, *C. elegans* was chosen as a model organism for two major reasons: (1) the mitochondrial structure and functions are mostly conserved across eukaryotes, including humans, and (2) the short life span and genetic tractability in worms allow for the study of whole-organism level physiological and longevity parameters in a convenient timeframe.

In the current study, we knocked down *psd-1* in *C. elegans* using RNA interference (RNAi), which led to severe phenotypes including shortened lifespan, developmental delay, reduced respiration capacity, accumulation of ROS, and DAF-16 nuclear translocation, an indicator of stress response. This stress response is mediated by protective signaling pathways, including the transcription factor DAF-16 (FOXO), a master regulator of the antioxidant defense system in *C. elegans.* Under normal conditions, DAF-16 remains inactive within the cytoplasm, but stress conditions trigger its nuclear translocation [17]. Then, DAF-16 activates the expression of downstream antioxidant and longevity-associated genes to combat oxidative damage. In *psd-1* knockdown worms, DAF-16 was translocated to the nucleus to activate antioxidant genes against oxidative stress. All these effects point to the fact that PE biosynthesis via PSD-1 is critical for mitochondrial activity and development of the organism. We hypothesized that depletion of *psd-1* in *C. elegans* causes two distinct failures—(1) structural and functional breakdown of the respiratory chain due to decreased PE levels, and (2) a signaling crisis triggered by oxidative stress. In the present study, we found that co-knockdown of key mitochondrial longevity gene *clk-1* (which encodes demethoxyubiquinone hydroxylase, the enzyme catalyzing the final step of CoQ9 biosynthesis) along with *psd-1* prevented developmental delay phenotype, significantly restored lifespan and oxygen consumption rate while maintaining normal ROS levels. This reveals a genetic interaction between these two pathways.

To understand the molecular mechanism of genetic rescue, it is important to know the existing biological functions of *clk-1*. CoQ9 is involved in transferring electrons from complex I and II to complex III in the electron transport chain. Mutations in this gene showed significantly increased lifespan, though worms exhibited abnormal development and behavior [18,19,20]. It has been demonstrated that modulating the *clk-1* gene directly controls respiration, behavior and longevity in worms by decreasing mitochondrial function [21]. Consistently, cultured embryonic cells from *Coq7*-deficient mice exhibit slow cell proliferation and delayed cell division. These results collectively indicate the importance of *clk-1/Coq7* in biosynthesis of Coenzyme Q, maintaining the mitochondrial integrity and neurogenesis in mice [22].

In summary, our work fills this gap by showing that disease phenotypes caused by phospholipid depletion can be uncoupled from the underlying structural lipid defect through modulation of the ubiquinone pathway. Taken together, our study highlights the relationship between mitochondrial phospholipid metabolism, ubiquinone pathway and organismal health. By identifying the fact that ETC-mediated retrograde signaling and ROS are the major causes of the observed pathology in the PE-deficient worms, our observations directly reveal the role of mitochondrial ROS in health and disease.

## 2. Materials and Methods

### 2.1. C. elegans Strains and Maintenance

All *C. elegans* strains were maintained at 20 °C on nematode growth media (NGM) agar plates seeded with *Escherichia coli* OP50 using the standard protocol [23]. All experiments were performed using age-synchronized population obtained by sodium hypochlorite (bleaching) treatment. After bleaching procedure, eggs were incubated overnight in M9 buffer on a shaker to get synchronized L1-stage worms. For developmental assays, synchronized L1 worms were transferred directly to experimental RNAi feeding plates. For all remaining experiments, L1 worms were allowed to develop on *HT115* (empty vector) and synchronized young adult stage worms were used. N2 (Bristol) strain was used as the wild-type control. N2 and DAF-16::GFP {Is[daf-16P::daf-16::GFP; rol-6(su1006)]} strains used in this work were procured from the Caenorhabditis Genetics Center (CGC, University of Minnesota, Minneapolis, MN, USA).

### 2.2. RNAi Treatment

RNAi experiments were performed using the RNAi *E. coli* clones obtained from the Ahringer *C. elegans* RNAi feeding library. These RNAi strains were cultured overnight at 37 °C in LB broth containing 50 µg/mL of carbenicillin. These overnight bacterial cultures were seeded onto the RNAi plates and incubated overnight at 37 °C. RNAi plates were prepared with IPTG at a final concentration of 1 mM and carbenicillin at a final concentration of 50 µg/mL. *HT115* bacterial strain carrying empty L4440 vector was used as a control in this study. For *C. elegans* single knockdown conditions (Control, *psd-1* (NCBI Gene ID-176027), *acs-22* (NCBI Gene ID-181138), *sod-3* (NCBI Gene ID-181748), *f15d3.6* (NCBI Gene ID-173040), *nhr-49* (NCBI Gene ID-172839) and *clk-1* (NCBI Gene ID-175729)), cultures were maintained at their original OD_600_ ~ 0.8 and for double knockdown conditions, equal density (OD_600_) suspensions of both the RNAi bacteria were mixed thoroughly in 1:1 (*v*/*v*) ratio before seeding. Final OD_600_ of double-knockdown mixed cultures was also maintained at OD_600_ of ~0.8 to ensure normalized knockdown efficiency across RNAi conditions.

### 2.3. Semi Quantitative PCR Analysis of psd-1 and clk-1 mRNA Expressions

Total cellular RNA was extracted from ~500 worms per replicate using an RNA extraction kit (HiMedia, Thane, India). Worms were lysed using liquid nitrogen followed by mortar pestle grinding, lysed worm samples were then processed for RNA extraction following the manufacturer’s protocol. cDNA was synthesized from 1 µg of total RNA per condition using a cDNA synthesis kit (Bio-Rad Laboratories, Hercules, CA, USA). cDNA-specific primers were used to amplify specific genes using PCR (see Table S1). PCR products were run on 4% agarose gel and visualized under a Bio-Rad ChemiDoc imaging system. Densitometric analysis of bands was measured by ImageLab software (version 6.1.0, build 7). Relative mRNA expressions of *psd-1* and *clk-1* were normalized against *act-1* expression levels from three independent experiments.

### 2.4. Developmental Assay

To evaluate the impact of single and double knockdowns on *C. elegans* growth, we performed a developmental assay comparing control (*HT115*), single (*psd-1* or *clk-1 RNAi*) and double (*psd-1*; *clk-1* RNAi). Development and body length of synchronized L1 worms were evaluated at every stage until they reached adulthood. Twenty worms per condition were then randomly selected, washed and fixed using 4% paraformaldehyde. Images were taken at 10× magnification using Optika brightfield microscope (Optika S.r.l., Bergamo, Italy). The experiment was conducted in three independent biological replicates. Growth and body length measurements were evaluated densitometrically using ImageJ software (Version 1.54). Statistical analysis was performed using GraphPad Prism (Version 6.0). Statistical significance for *psd-1* RNAi vs. control was measured using Welch’s *t* test and comparisons among multiple groups were carried out using one-way ANOVA. Graphs were plotted using GraphPad Prism.

### 2.5. Ethanolamine Supplementation

For rescue experiments, ethanolamine was dissolved in sterile deionized water to prepare a concentrated stock solution. The stock solution was then filtered and added to the NGM agar media at a final working concentration of 5 mM [24]. Synchronized L1 larvae were transferred onto these ethanolamine-supplemented control and RNAi feeding plates. 20 worms per condition were monitored and imaged at 10× magnification using an Optika brightfield microscope. Body lengths were analyzed using ImageJ software, following the exact parameters given in Section 2.4. Experiment was conducted in three independent biological replicates and representative images of one experiment were shown. Analysis and graphs were carried out using GraphPad Prism.

### 2.6. Lifespan Assay

Lifespan assays were performed on NGM (containing IPTG and carbenicillin) and FIC plates (NGM supplemented with 50 µM FUDR (fluorodeoxyuridine) to inhibit egg hatching). Age-synchronized young adult worms (100 worms per condition) were transferred onto control and RNAi feeding plates. On every alternative day, live worms were counted and transferred to new plates, dead ones were confirmed by gentle stimulation using a platinum wire picker, and worms that showed internal hatching, bagging, or which were crawling off the agar surface were considered censored. Kaplan–Meier survival analysis of knockdown worms was compared with control worms using the Log-Rank test, with a Bonferroni-corrected *p*-value of <0.0001. Statistical analysis of survival curves was performed using OASIS2 software [25]. The experiment was performed three times independently and *p*-values were determined from Chi-square test.

### 2.7. Brood Size Assay

To determine the effect of knockdown on reproductive health of the organism, we performed brood size assay of control and knockdown worms. Ten young adult worms per condition grown on *HT115* were transferred into experiment plates. Each worm was allowed to lay eggs and was then transferred to a new plate every 12 h until they stopped laying eggs. The eggs that were laid by each worm were counted and scored. The experiment was performed three times. Statistical significance for *psd-1* RNAi vs. control worms was measured by Welch’s *t* test and graphs were plotted using GraphPad Prism software.

### 2.8. Measurement of Oxygen Consumption Rate (OCR) in C. elegans

Oxygen consumption rate (OCR) was measured using a Clark-type oxygen electrode system (Oxytherm+, Hansatech Instruments, King’s Lynn, UK). Age-synchronized adult stage worms were collected directly from RNAi feeding plates without starvation and were quickly washed three times with M9 buffer to remove any residual bacteria to reflect normal physiological respiration. For each biological replicate, approximately 5000 worms in 500 µL M9 buffer were added to the Oxytherm+ respiration chamber containing another 500 µL M9 buffer for the final volume of 1 mL in the chamber. The chamber temperature was maintained and calibrated at 20 °C and the samples were stirred continuously throughout the experiment. Dissolved oxygen concentration was recorded continuously for 10 min after sample loading using OxyTrace+ software (V1.0.48) (Hansatech Instruments, Norfolk, UK). The oxygen consumption rate was obtained from the decrease in the oxygen levels over time and expressed in nmol O_2_/min/5000 worms. The worms were also counted post-measurement. Each experimental condition was conducted in three independent biological replicates and *p*-values were determined using one-way ANOVA.

### 2.9. Measurement of Reactive Oxygen Species (ROS)

Intracellular ROS levels were measured using 2′,7′-dichlorodihydrofluorescein diacetate (DCFH-DA) probe [26]. Briefly, age-synchronized adult worms from control, *psd-1* single knockdown and *psd-1*; *clk-1* double knockdown groups were collected and washed three times with M9 buffer (pH 7.0) to eliminate residual and gut bacteria. All assays were performed in M9 buffer to lower pH-driven changes due to DCFH-DA. Thirty worms from each condition were picked and incubated with DCFH-DA at a final concentration of 50 µM in the black 96-well plate in triplicates; 3% (*v*/*v*) hydrogen peroxide (H_2_O_2_) was used as a positive control. Final volume in each well was adjusted to 150 µL using M9 buffer. Fluorescence intensity was measured using a Variaskan ALF multimode microplate reader (Thermo Fisher Scientific, Waltham, MA, USA) with excitation and emission wavelengths of 504 nm and 525 nm, respectively, at every 1 h interval for a total duration of 12 h. The sample fluorescence values were background corrected and normalized to the total number of worms taken in each well. The experiment was conducted in three independent biological replicates and *p*-values were determined using two-way ANOVA with Tukey post hoc analysis.

### 2.10. Daf-16: GFP Nuclear Localization Assay

To monitor the subcellular translocation of the DAF-16, we used DAF-16::GFP reporter strain (Is[daf-16P::daf-16::GFP; rol-6(su1006)]) in this experiment. Worms were cultured on *HT115* bacteria until they reached young adult stage at 20 °C. Then, age-synchronized young adult worms were transferred to RNAi feeding plates containing *HT115*, *psd-1*, *clk-1*, and *psd-1*; *clk-1* bacterial clones at 20 °C. After 48 h, worms were harvested and anesthetized using 20 mM levamisole in M9 buffer and mounted on the slides with 2% (*w*/*v*) agarose pads. Images were captured at 10× magnification using Optika fluorescence microscope. The distribution of fluorescence signals within nucleus and cytoplasm was quantified using ImageLab software. Thirty worms per condition were selected randomly for imaging. The experiment was conducted in three independent biological replicates. The worms were categorized into cytoplasmic and nuclear localization groups, and graphs were plotted using GraphPad Prism.

### 2.11. Body Fat Analysis Using Nile Red

Total body fat content was measured using Nile red staining (Thermo Fisher Scientific, Waltham, MA, USA). Nile red stocks of 5 mg/mL concentration were prepared by dissolving Nile red powder in acetone and stored at −20 °C protected from light. As mentioned earlier, age-synchronized young adult stage worms were transferred to RNAi feeding plates. The plates were incubated for 48 h at 20 °C. Worms were then washed with M9 buffer three times and fixed by treating with 40% isopropanol for 3 min at 20 °C. Fixed worms were centrifuged at 560× *g* for 30 s and supernatant was discarded. Nile red working solution (30 µg/mL in M9 buffer) was added to worms and incubated for 2 h in dark at 20 °C. After incubation, worms were washed 2–3 times with M9 buffer and mounted on the slides with 2% (*w*/*v*) agarose pads. Fluorescence images of 22 worms per condition were captured using a fluorescence microscope and relative fluorescence intensity was quantified using ImageJ software. The experiment was conducted in three independent biological replicates and *p*-values were determined by two-way ANOVA with Tukey post-hoc analysis using GraphPad Prism.

### 2.12. Mitochondria Isolation

Crude mitochondria were isolated using differential centrifugation from the previously described protocol with slight modifications [27]. Age-synchronized adult stage worms from six 90 mm RNAi feeding plates (approximately >200,000 worms) per each condition were harvested to isolate *C. elegans* mitochondria. Worms were collected, washed with M9 buffer by centrifuging at 1000× *g* for 1 min and supernatant was discarded. Then, 10 mL of M9 buffer was added to the worm pellet and rotated for 60 min to remove bacteria from intestinal tract. Worms were washed twice with M9 at 200× *g* for 1 min to remove residual bacteria, supernatant was removed and pellet was placed on ice. The final worm pellet was resuspended in 5 mL of ice-cold sucrose buffer (1 M Sucrose, 0.1 M Tris/MOPS pH 7.4, 0.1 M EDTA/Tris pH 7.4), centrifuged gently for 1 min, supernatant removed. Pellet was resuspended in 1 mL ice-cold sucrose buffer and transferred to the pre-chilled Dounce homogenizer, and 100 strokes were performed with the B ‘tight’ pestle on ice. Homogenate was centrifuged at 2000× *g* for 5 min at 4 °C to pellet broken worm bodies and cell debris. Then, mitochondria-containing supernatant was transferred into a pre-chilled new tube and centrifuged at high speed at 13,000× *g* for 20 min at 4 °C. Final mitochondrial pellet was gently resuspended in an ice-cold sucrose buffer and stored at −80 °C. The experiment was conducted in three independent biological replicates. Mitochondrial protein concentration was determined using Bradford assay [28,29].

### 2.13. Mitochondrial Phospholipid Extraction

Total mitochondrial lipids were extracted from three independent mitochondrial isolations using the Folch method [30]. Briefly, before extraction, total mitochondrial protein concentration was measured using Bradford assay. Approximately 80 µg of mitochondrial protein from each condition was used to extract lipids. Mitochondrial samples were resuspended in 3 mL ice-cold chloroform-methanol (1:2, *v*/*v*) and vortexed vigorously for 1 min at maximum speed. Deionized water was then added at 0.2 × volume, gently vortexed for 15 s and centrifuged at 1000× *g* for 2 min at 4 °C. The bottom organic layer was transferred to a new tube. To this, an equal mixture of methanol:water (1:1), supplemented with 0.01% BHT, was added at 0.2 × volume of organic phase, followed by vortexing for 15 s. This was centrifuged at 1000× *g* for 2 min and the bottom layer was again transferred to a fresh tube. This methanol:water (1:1) wash step was repeated three times, and the final bottom organic layer was collected in a glass vial. Solvent was evaporated under vacuum using a rotary evaporator (SpeedVac concentrator, Thermo Fisher Scientific, Waltham, MA, USA) and nitrogen gas. Dried lipids were stored at −80 °C until use.

### 2.14. TLC and Phospholipid Quantification

Phospholipids from extracted lipids were separated and quantified on TLC silica gel 60 (Sigma-Aldrich, St. Louis, MO, USA) using TLC [31]. Prior to the experiment, TLC plates were activated by inserting the plates in 1.8% (*w*/*v*) boric acid for 2 min and dried in an oven at 110 °C for 30 min. Meanwhile, a glass TLC chamber was equilibrated using mobile phase consisting of chloroform:ethanol:water:triethylamine (30:35:7:35). Each sample was spotted in each lane and air-dried for 2 min. The spotted TLC plate was placed into a saturated TLC chamber containing solvent mixture and let run until solvent front reached 1 cm from top of the plate. Then, plate was removed and air-dried for 5 min. Plates were then stained with cupric sulfate (CuSO_4_) solution for band visualization and air-dried completely for 30–40 min. Plate was then placed on the hot plate at 180 °C for 5 min until bands appear. Phospholipid standards from Avanti Polar Lipids (Alabaster, AL, USA), were used as controls. Phospholipid bands were quantified using ImageLab software. The experiment was conducted in three independent biological replicates. *p*-values were determined by Welch’s *t* test and graphs were plotted using GraphPad Prism.

### 2.15. Quinone Extraction and High-Performance Liquid Chromatography (HPLC)

Quinones were extracted and quantified by high-performance liquid chromatography (HPLC) using the procedure described previously [32]. Briefly, 5000 worms for each condition were washed with M9 buffer four times to remove residual bacteria. 1 mL of water with 50 μL of 2,6-di-tert-butyl-4-methyl-phenol (BHT) (10 mg/mL in ethanol) was added to the worm pellet to minimize oxidative degradation during sample processing and homogenized using a Dounce homogenizer (Kimble, Millville, NJ, USA). Then, 100 µL of lysate was taken aside to measure protein concentration by Bradford assay; 1 mL of 0.1 M SDS was added to the lysate and further homogenized. Next, 2 mL of ethanol was added to the lysate and vortexed for 30 s. Then, 2 mL of hexane was added, vortexed and centrifuged at 1000× *g* for 5 min at 4 °C to separate layers. The upper organic layer was collected in a new tube, and hexane extraction was repeated. Both upper phases were combined and evaporated using a SpeedVac concentrator (Thermo Fisher Scientific, Waltham, MA, USA). Samples were stored at −80 °C until use. Samples were reconstituted in 100 µL mobile phase (70% methanol and 30% ethanol) and injected into a reverse-phase C18 column (2.1 mm × 150 mm, 3.5 µm) at flow rate of 0.500 mL/min. Peaks were detected using DAD detector (Agilent Technologies, Inc., Santa Clara, CA, USA) at 275 nm. As pure DMQ9 standard is not commercially available, DMQ9 and CoQ9 peaks were assigned based on retention time relative to the CoQ10 external standard, aligning with established *C. elegans* quinone profiling literature. These peaks were consistent with the expected elution order for these related ubiquinones on reverse-phase chromatography. DMQ9 eluted slightly earlier than CoQ9, which aligns with its lower polarity. This assignment was primarily supported by the reproducible retention-time across all the samples and replicates. This aligns with the known role of CLK-1 in converting DMQ9 to CoQ9. DMQ9 and CoQ9 standards were not available for co-injection confirmation in this study. Thus, peak assignments should be considered as tentative until it is confirmed by LC-MS/MS in future work. Concentrations were estimated by comparing the known concentrations and peak areas of standards. A single external calibration curve of CoQ10 standards was used to quantify CoQ9 and DMQ9, as they share a common benzoquinone (chromophore) across all ubiquinone species, producing comparable UV absorptivity at 275 nm. This approach was followed from the previous *C. elegans* studies that quantified multiple ubiquinone species (CoQ9, CoQ10, DMQ9, CoQ8) against a single standard [33]. Data was normalized to protein content, and results were expressed as pmol/mg protein. Experiment was conducted in three independent biological replicates. Statistical analysis was carried out using one-way ANOVA with Tukey post hoc analysis and graphs were plotted using GraphPad Prism.

### 2.16. Statistical Analysis

All statistical analyses were performed using GraphPad Prism software. For pairwise comparisons between control and single-knockdown conditions, statistical significance was assessed using Welch’s *t*-test. For comparisons among three or more groups, one-way ANOVA followed by Tukey’s post-hoc test was used. Experiments such as Nile red and cellular ROS, which are time-coursed, were analyzed by two-way ANOVA with Tukey’s multiple comparisons test. Lifespan data were analyzed by Kaplan–Meier survival analysis using the Log-rank test with Bonferroni correction and significance was calculated by Chi-square test using OASIS2 software. All experiments were performed in three independent biological replicates unless otherwise stated, and data are presented as mean ± SD. A *p*-value of <0.05 was considered statistically significant (* *p* < 0.05, ** *p* < 0.01, *** *p* < 0.001, **** *p* < 0.0001).

## 3. Results

### 3.1. PSD-1 Deficiency Results in Developmental Delay, Reduced Lifespan, Impaired Reproduction

**Figure 1:**
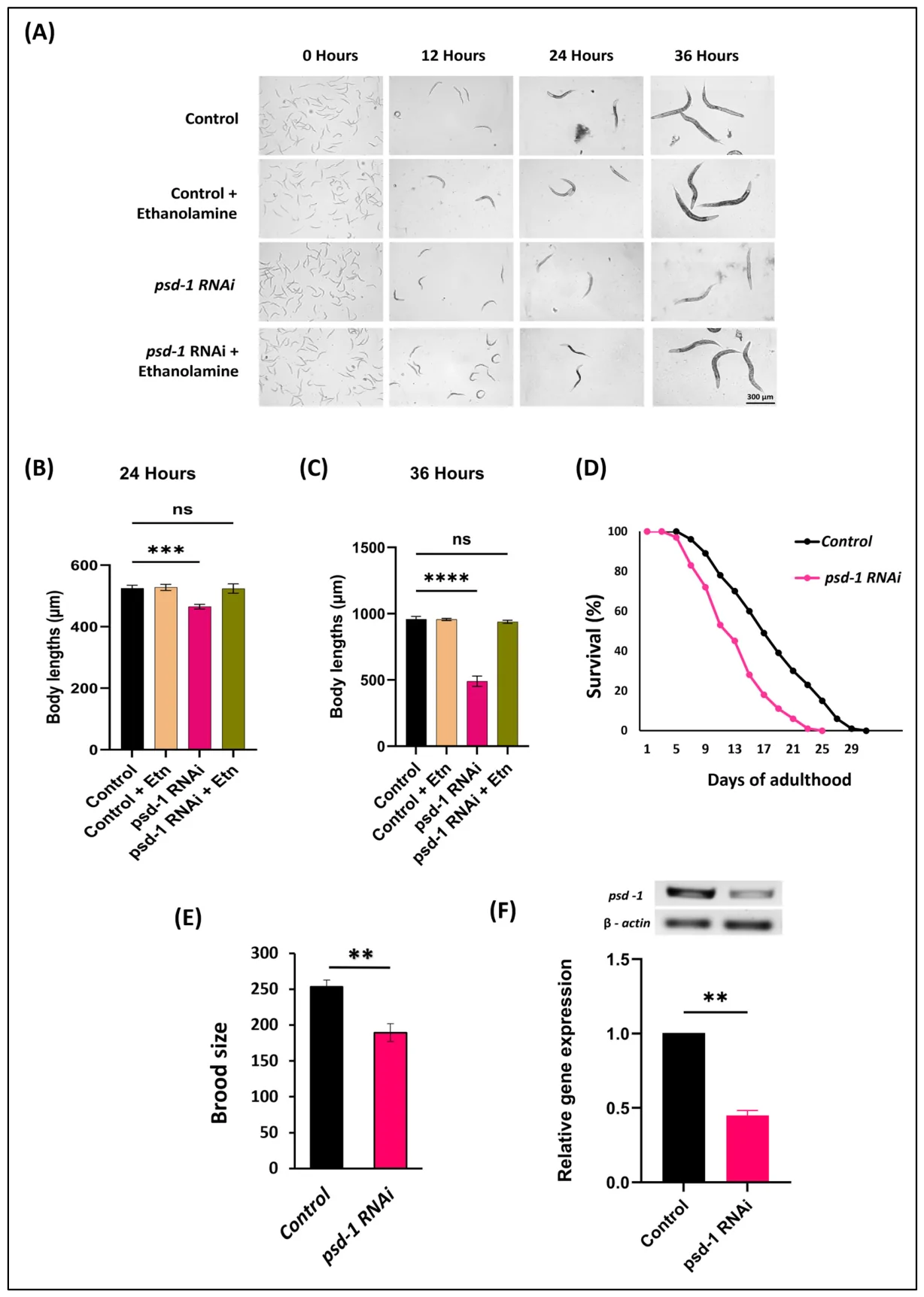
(**A**) Comparative images of *C. elegans* reveal a developmental delay in *psd-1* knockdown animals relative to control worms fed with HT115 (EV, empty vector) rescued by ethanolamine supplementation. Control worms reached young adult stage while *psd-1* knockdown worms stayed in earlier larval stages, suggesting delay in development. Images were taken every 12 h under the same magnification. Scale bar = 300 µm. (**B**) Mean of body length (µm) at 24 h of control, *psd-1* RNAi, control + ethanolamine, and *psd-1* RNAi + ethanolamine worms (*n* = 20 worms per condition). Data are presented as mean ± SD (*** *p* < 0.001, one-way ANOVA). (**C**) Mean body length (µm) of control, *psd-1* RNAi, control + ethanolamine, and *psd-1* RNAi + ethanolamine worms (*n* = 20 worms per condition) at 36 h. (**D**) Lifespan analysis of HT115 control, *psd-1* RNAi worms (*n* = 100 worms per condition). Kaplan–Meier survival curve of lifespan assay showed *psd-1* knockdown worms have a noticeably shorter lifespan than wild-type controls. The mean lifespan of control worms was 18.37 ± 0.64 days, whereas *psd-1* RNAi worms exhibited a reduced mean lifespan of 13.48 ± 0.51 days. Lifespan values were expressed as mean ± SE. The median lifespans (50% mortality) were 19 days and 13 days, respectively. (**E**) Brood size analysis revealed a significant reduction in the total number of progenies produced by *psd-1* knockdown worms compared to control worms (*n* = 10 worms per condition), indicating impaired reproductive capacity. (**F**) Semi-quantitative RT-PCR validation of *psd-1* knockdown efficiency following RNAi-mediated knockdown, normalized to *act-1*. All the experiments were conducted in three independent biological replicates. Data are presented as mean ± SD (** *p* < 0.01, *** *p* < 0.001 and **** *p* < 0.0001, ns = not significant).

To investigate the physiological role of PSD-1 in *C. elegans*, we generated *psd-1* knockdown worms using RNAi feeding method. Synchronized *psd-1* knockdown worms showed significant delay in development when compared to control worms (*HT115*) (Figure 1A). Control worms reached young adult stage while *psd-1* RNAi worms remained in L2/L3 stage, indicating delay in growth. The mean body length of *psd-1* knockdown worms showed small body size at 24 h (*p* = 0.0008) and 36 h (*p* < 0.0001) (Figure 1B,C). Also, to determine whether the physiological effects caused by PSD-1 loss were due to reduced PE levels, we supplemented exogenous ethanolamine (5 mM) to the control and *psd-1* knockdown worms. Ethanolamine supplementation significantly rescued development delay in *psd-1* knockdown worms (Figure 1A–C). *psd-1* RNAi, when supplemented with ethanolamine, showed rescued development (*psd-1* RNAi vs. *psd-1* RNAi + ethanolamine, *p* = 0.0009 at 24 h and *p* < 0.0001 after 36 h of development). This chemical rescue provides functional validation that the physiological defects seen in *psd-1* knockdown worms are due to the collapse of PE homeostasis.

Simultaneously, we performed lifespan analysis to see the effect of *psd-1* loss which revealed that *psd-1* knockdown significantly shortened lifespan compared to wild-type controls (*p* < 0.0003) (Figure 1D). The mean lifespan of *psd-1* RNAi worms was reduced to 13.48 ± 0.51 days, as compared to 18.37 ± 0.64 days in control worms. This corresponds to the decrease of median lifespan from 19 days in control worms to 13 days in *psd-1* knockdown worms. Furthermore, to check the reproductive capacity of these worms, we quantified total progeny using standard brood size assay. Results showed significant reduction in egg production by knockdown worms compared to controls (*p* = 0.0020) (Figure 1E). To validate knockdown efficiency, we analyzed *psd-1* mRNA expression, which was significantly reduced in *psd-1* RNAi worms (*p* = 0.0013) (Figure 1F). These results highlight the requirement of *psd-1*-mediated lipid biosynthesis for normal growth, development, aging, and reproduction.

### 3.2. Knockdown of psd-1 Reduces Mitochondrial Phosphatidylethanolamine (PE) Levels

**Figure 2:**
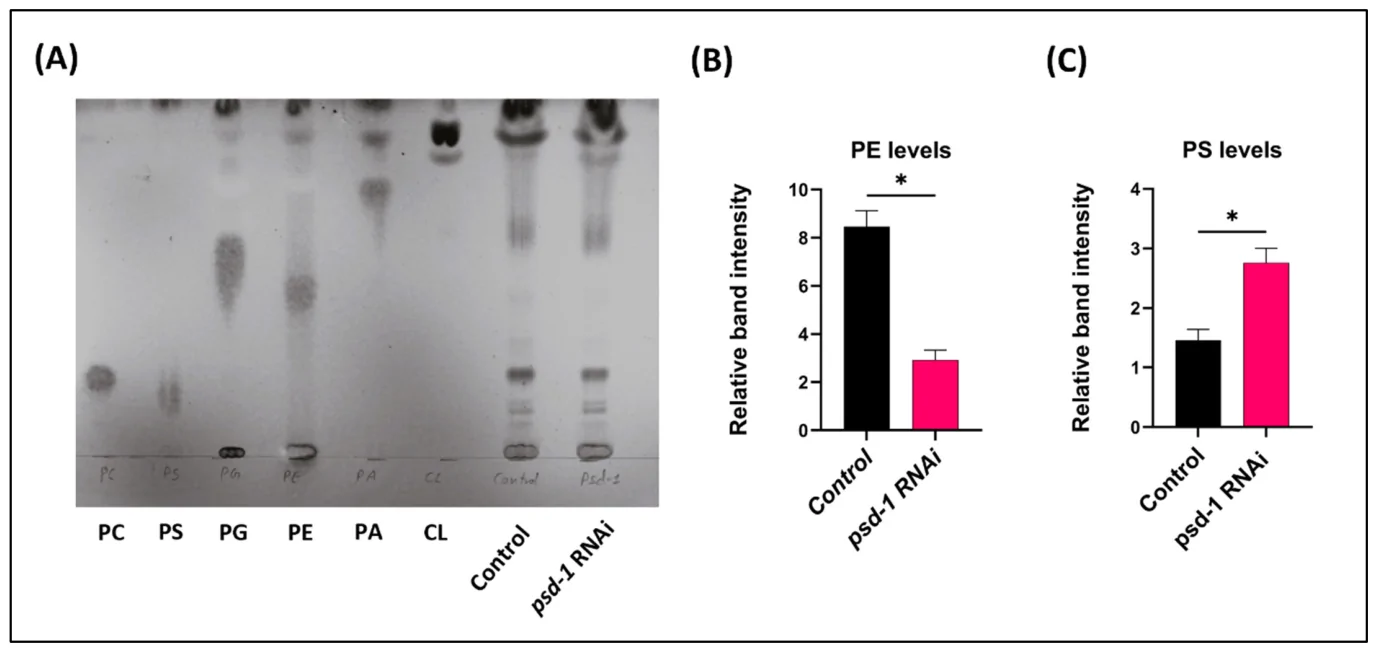
Separation of mitochondrial phospholipids and quantification using thin-layer chromatography. (**A**) Thin-layer chromatography for phospholipid separation. Lane 1–Lane 6 are phospholipid standards. Lane 7: mitochondrial phospholipids extracted from control worms; Lane 8: mitochondrial phospholipids extracted from *psd-1* RNAi worms. (**B**) Densitometric analysis of PE levels from TLC bands, showing reduction in PE in *psd-1* knockdown worms. (**C**) Relative levels of phosphatidylserine (PS) in control and *psd-1* knockdown worms. Phospholipids were extracted from three independent mitochondrial isolations (*n* = 3 biological replicates per group). Data are presented as mean ± SD; statistical significance was determined using an unpaired two-tailed Student’s *t*-test (* *p* < 0.05).

To verify whether these observed physiological effects were connected to reduced phospholipid homeostasis, we quantified mitochondrial phosphatidylethanolamine (PE) levels in *psd-1* knockdown worms. Crude mitochondria were isolated from age-synchronized young adult worms, extracted lipids and separated phospholipids using TLC. There is a significant decrease in PE levels in *psd-1* knockdown worms compared to control (Figure 2A). Densitometric analysis of the PE band in the TLC confirmed the substantial reduction in mitochondrial PE levels (*p* = 0.0171) (Figure 2B). Intriguingly, increased levels of PS were observed in *psd-1* knockdown worms, indicating the accumulation of primary substrate PS (*p* = 0.0313) (Figure 2C). Because PSD-1 directly decarboxylates PS to produce PE in mitochondria, this substrate-product shift provides strong validation for successful PSD-1 inhibition. Collectively, these results confirm that PSD-1 is the major contributor to the mitochondrial PE pool in *C. elegans,* and also that its depletion affects mitochondrial lipid homeostasis.

### 3.3. Co-Knockdown of psd-1 with clk-1 Prevents Developmental Delay Phenotype and Restores Lifespan

**Figure 3:**
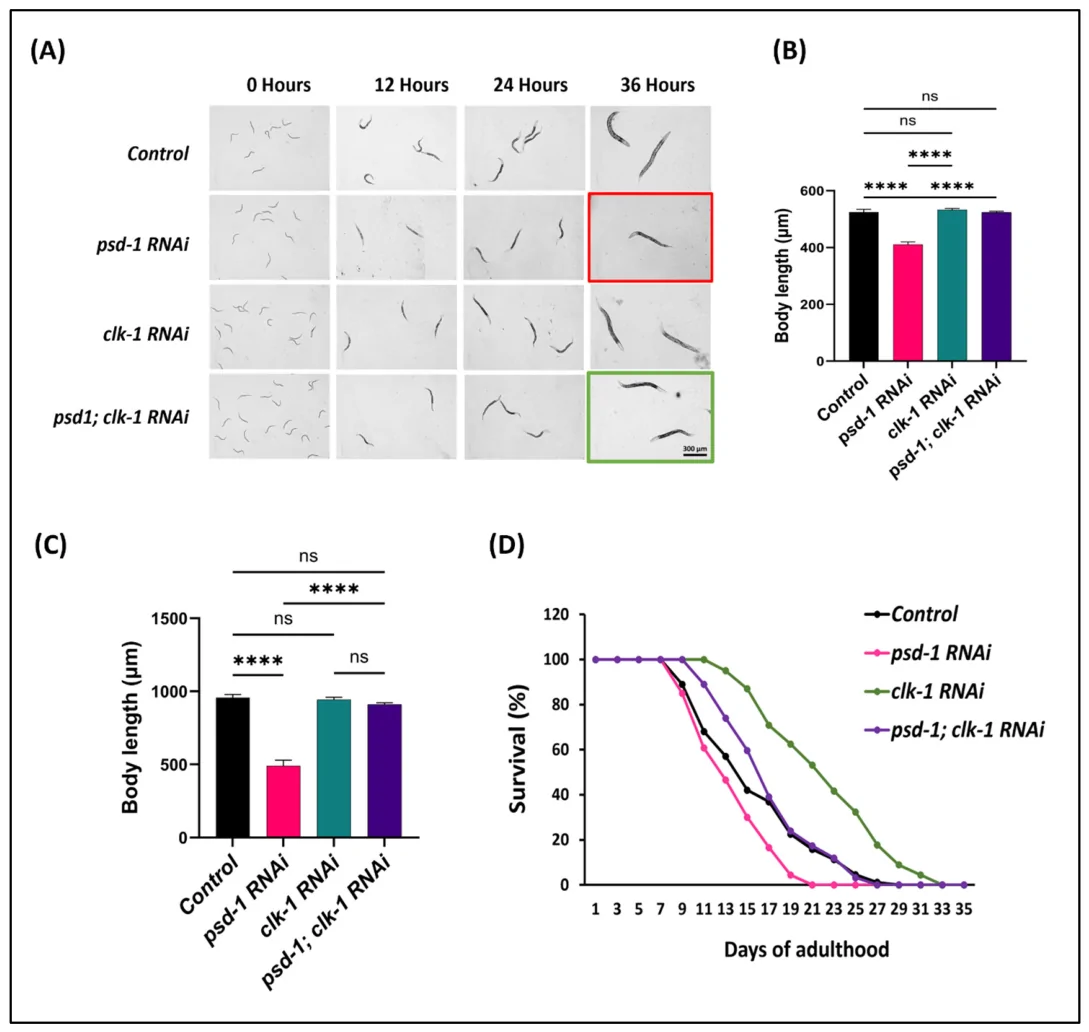
Co-knockdown of *psd-1* and *clk-1* rescued developmental delay and restored lifespan. (**A**) Comparative brightfield images of control, *psd-1* knockdown and *psd-1*; *clk-1* double knockdown worms. Developmental delay that was seen in *psd-1* knockdown animals was rescued in double knockdown worms. Images were taken every 12 h under the same magnification. Scale bar = 200 µm. The red box indicates developmental delay, and the green box indicates rescue. (**B**) Mean body length of control, *psd-1* knockdown worms, *psd-1*; *clk-1* double knockdown worms at 24 h (*n* = 20 worms per condition). (**C**) Mean body length of control, *psd-1* knockdown worms, *psd-1*; *clk-1* double knockdown worms at 36 h (*n* = 20 worms per condition). Data are presented as mean ± SD (**** *p* < 0.0001, one-way ANOVA), ns = not significant. (**D**) Lifespan analysis of HT115 control, *psd-1* RNAi worms, *clk-1* RNAi and *psd-1*; *clk-1* RNAi (*n* = 100 worms per condition). Kaplan–Meier survival curve shows restored lifespan in the *psd-1*; *clk-1* double knockdown relative to *psd-1* single knockdown. Mean lifespans were 16.03 ± 0.56 days for control, 13.86 ± 0.36 days for *psd-1* RNAi, 22.47 ± 0.56 days for *clk-1* RNAi, and 17.36 ± 0.44 days for *psd-1*; *clk-1* RNAi. The corresponding median lifespans (50% mortality) were 15, 13, 23, and 17 days, respectively. Lifespan values were expressed as mean ± SE. All the experiments were performed in three independent biological replicates.

To investigate genetic interaction between *psd-1* and genes representing distinct pathways such as intramitochondrial PS transport (*f15d3.6/prel*-3), fatty acid metabolism (*acs*-22), transcriptional regulator of lipid homeostasis (*nhr*-49), mitochondrial oxidative stress (*sod*-3) and mitochondrial ETC/CoQ metabolism (*clk-1*—ubiquinone biosynthesis gene), we performed double knockdown of *psd-1* along with *f15d3.6, sod-3, acs-22, nhr-49* and *clk-1* (Supplementary Figure S1). Of all the double knockdowns, *psd-1*; *clk-1* double worms showed rescued development (Figure 3A). This suggests that the co-knockdown of *clk-1* with *psd-1* rescues the developmental delay phenotype caused by *psd-1* depletion. Mean body length measurements of *psd-1*; *clk-1* RNAi showed normal development at 24 h (*p* < 0.0001) and 36 h (*p* < 0.0001) (Figure 3B,C).

In addition to rescuing development of an organism, co-knockdown of *clk-1* also significantly restored the lifespan phenotype in *psd-1*-deficient worms (*psd-1*; *clk-1* RNAi vs. *psd-1* RNAi; *p* < 0.0001) (Figure 3D). Interestingly, double knockdown showed a mean lifespan of 17.36 ± 0.44 days, partially rescuing the shortened lifespan phenotype observed in *psd-1* single knockdown worms. Conversely, *clk-1* RNAi significantly extended the mean lifespan to 22.47 ± 0.56 days. The restoration of both development and lifespan in the double knockdown suggests that the adverse consequences of *psd-1* reduction can be mitigated by decreased CLK-1 activity. Taken together, these results reveal a significant genetic interaction between the *psd-1* and *clk-1.* These findings also suggest that defects caused by phospholipid depletion are not irreversible or permanent, but they can be decoupled by targeted genetic modification of the ubiquinone pathway.

### 3.4. Functional Validation of clk-1 Knockdown and Quinone Profiling in Knockdown Worms

**Figure 4:**
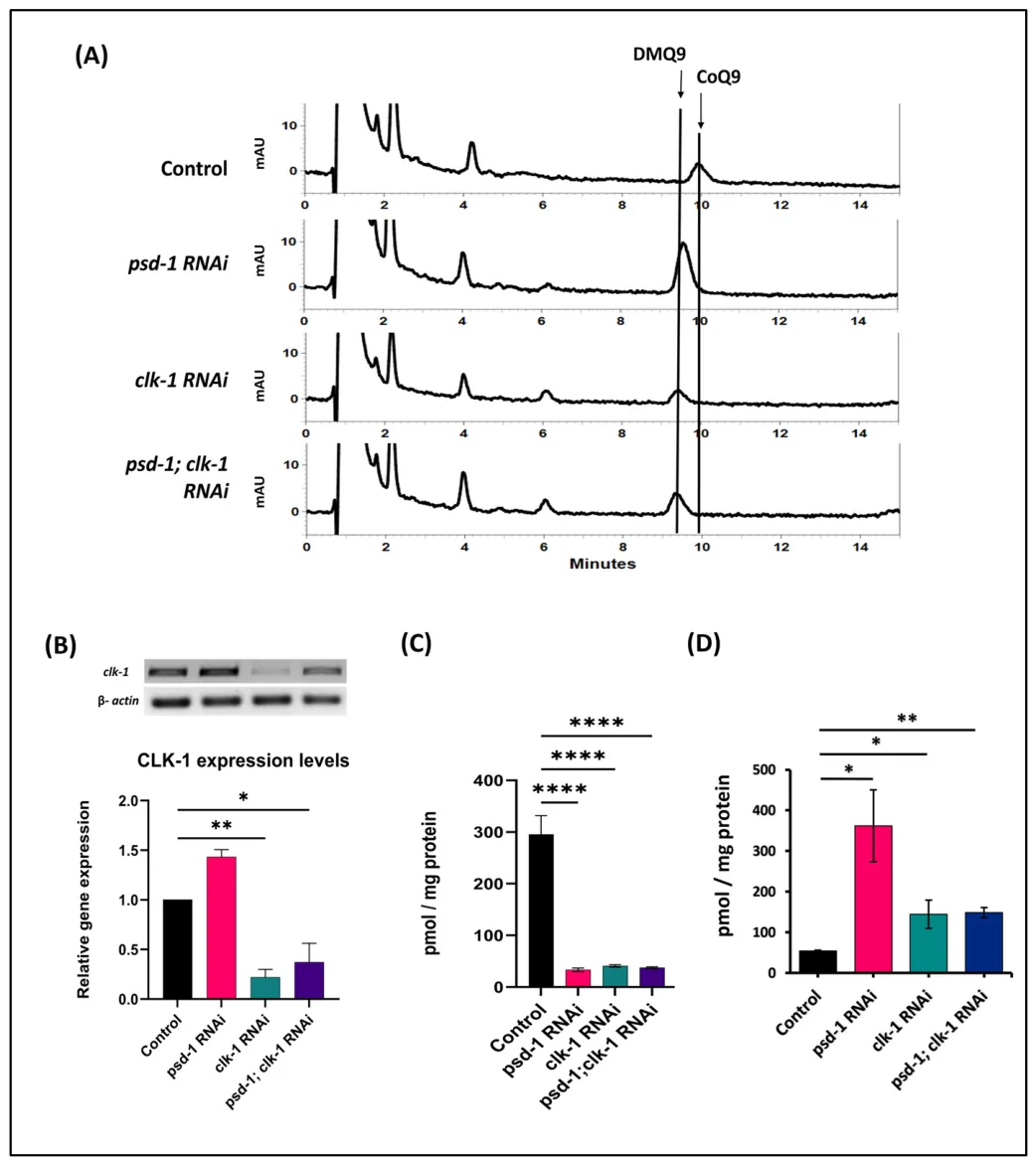
Functional validation of *clk-1* knockdown in knockdown worms. (**A**) clk-1 mRNA expression levels. (**B**) Chromatograms of high-performance liquid chromatography (HPLC) profiling of CoQ9 levels across control, *psd-1* knockdown, *clk-1* knockdown, and *psd-1*; *clk-1* double knockdown worm samples. (**C**) Quantification of Coenzyme Q9 (CoQ9). (**D**) Quantification of demethoxyubiquinone (DMQ9) levels showing accumulation of the precursor in *clk-1* single and double knockdown backgrounds. Error bars represent mean ± SD from *n* = 3 independent biological replicates. (* *p* < 0.05, ** *p* < 0.01, and **** *p* < 0.0001; one way ANOVA).

mRNA expression levels of *clk-1* were analyzed to validate knockdown efficiency in control, *psd-1* RNAi, *clk-1* RNAi and *psd-1*; *clk-1* RNAi worms. Results showed significant reduction in *clk-1* expression in *clk-1 RNAi* and *psd-1*; *clk-1 RNAi* double knockdown worms (control vs. clk-1 RNAi; *p* = 0.0046 and control vs. *psd-1*; clk-1 RNAi; *p* = 0.0102). Surprisingly, *clk-1* expression was increased in *psd-1* single knockdown worms (control vs. *psd-1 RNAi*; *p* = 0.0387) (Figure 4A). To confirm the loss of CLK-1 activity in addition to knockdown, we performed quinone profiling of control, *psd-1* knockdown, *clk-1* knockdown and *psd-1*; *clk-1* double knockdown worm samples using HPLC. CLK-1 is involved in the hydroxylation of demethoxyubiquinone (DMQ9) to form ubiquinone (CoQ9). It is an important step in the biosynthesis of CoQ9. Loss of CLK-1 activity is expected to decrease CoQ9 and accumulate DMQ9 [32]. Consistent with this data, *clk-1* knockdown worms showed reduced levels of CoQ9 (*p* < 0.0001) and accumulation of DMQ9 compared to control (*p* = 0.0466). Surprisingly, the same pattern was observed in *psd-1* single knockdown and *psd-1*; *clk-1* double knockdown worms, CoQ9 levels were decreased (control vs. *psd-1* RNAi; *p* < 0.0001 and control vs. *psd-1*; *clk-1* RNAi; *p* = 0.0046) (Figure 4B,C) and DMQ9 levels were elevated (control vs. *psd-1* RNAi; *p* = 0.0265 and control vs. *psd-1*; *clk-1* RNAi; *p* = 0.0061) (Figure 4D). Data of CoQ10 standards were provided in Supplementary Figure S2. This confirms that *clk-1* knockdown efficiency was preserved in double knockdown worms. Taken together, RNA level validation and enzymatic level validation results confirm the decrease in the activity of CLK-1 in this study.

### 3.5. Co-Knockdown of psd-1 and clk-1 Partially Restores Mitochondrial Respiration Defects and ROS Levels Caused by psd-1 Depletion

**Figure 5:**
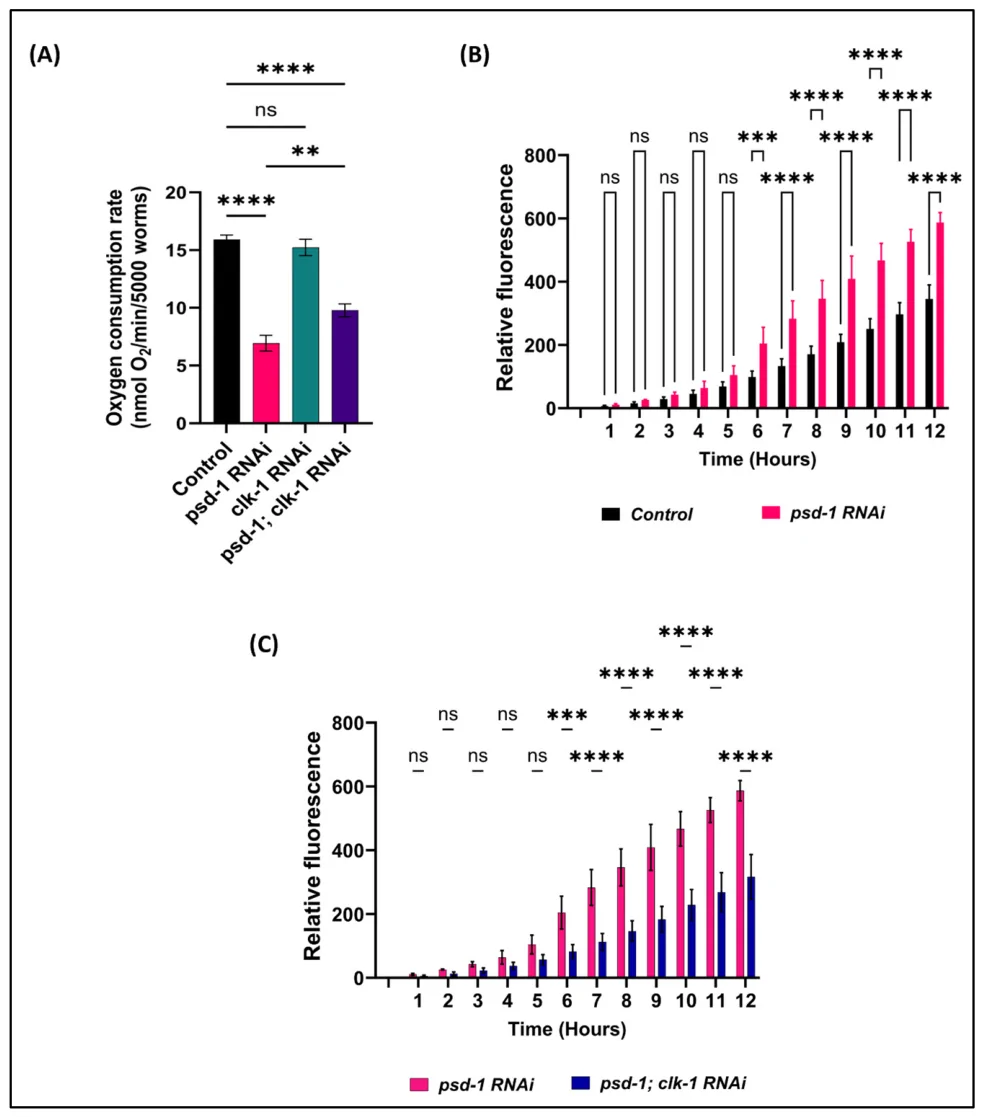
Co-knockdown of *psd-1* and *clk-1* partially restores mitochondrial respiration and decreases ROS levels. (**A**) Oxygen consumption rate (OCR) was measured in control, *psd-1, clk-1 and psd-1*; *clk-1* double knockdown worms and data was expressed as nmol O_2_/min/5000 worms. Data represent three independent biological experiments and are presented as mean ± SD (** *p* < 0.01, one-way ANOVA). (**B**) Intracellular ROS levels were measured across the same four conditions. ROS levels significantly increased in *psd-1* RNAi worms. (**C**) *psd-1*; *clk-1* double knockdown worms showed reduced ROS compared to *psd-1* knockdown worms. Fluorescence intensities were quantified in all the four conditions (*n* = 30 worms per condition). Data represent mean ± SD from *n* = 3 biological experiments; statistical significance was determined by two-way ANOVA with Tukey’s multiple comparisons test (*** *p* < 0.001, **** *p* < 0.0001, ns = not significant).

Since phosphatidylethanolamine plays an important role in maintaining the integrity of mitochondrial membrane and respiratory complexes, we hypothesized PE depletion would have a detrimental effect on mitochondrial respiratory function. Changes in membrane phospholipid composition can lead to increased electron leakage and poor electron transfer, thereby affecting ETC activity by increased ROS generation [34]. To evaluate this, we measured basal oxygen consumption rates (OCR) in control, *psd-1 RNAi*, *clk-1 RNAi* and *psd-1*; *clk-1 RNAi* worms using a Clark-type oxygen electrode system. The *psd-1*-deficient worms showed significantly reduced oxygen consumption compared to control worms (*p* = 0.0013) indicating severe impairment of mitochondrial respiration due to the decreased PE levels. The oxygen consumption rate of *clk-1* knockdown worms was similar to the control worms (*p* = 0.8935). On the contrary, oxygen consumption rate of *psd-1*; *clk-1* double knockdown worms was significantly increased compared to *psd-1* knockdown (*psd-1* RNAi vs. *psd-1*; *clk-1* RNAi; *p* = 0.0017) (Figure 5A).

Considering the fact that mitochondrial dysfunction is commonly associated with oxidative stress, we next measured intracellular reactive oxygen species (ROS) levels in all the conditions using DCFH-DA assay. An increase in ROS levels was observed in *psd-1* knockdown worms from the 6th hour when compared to controls (*p* < 0.0001). We also measured ROS levels in *clk-1 RNAi* and *psd-1*; *clk-1* double knockdown worms. As expected, *clk-1 RNAi* worms showed elevated levels of ROS compared to control worms from the 9th hour (*p* = 0.0092) (Supplementary Figure S3A,B). Unexpectedly, double knockdown worms showed lower ROS accumulation compared to that observed in *psd-1* knockdown worms (*psd-1* RNAi vs. *psd-1*; *clk-1* RNAi; *p* < 0.0001) (Figure 5B). These results suggest that downregulating CLK-1 activity in a PE-deficient background reduces oxidative stress.

### 3.6. Co-Knockdown of psd-1 and clk-1 Restores DAF-16 to the Cytoplasm Concomitant with Rescue of Physiological Phenotypes

**Figure 6:**
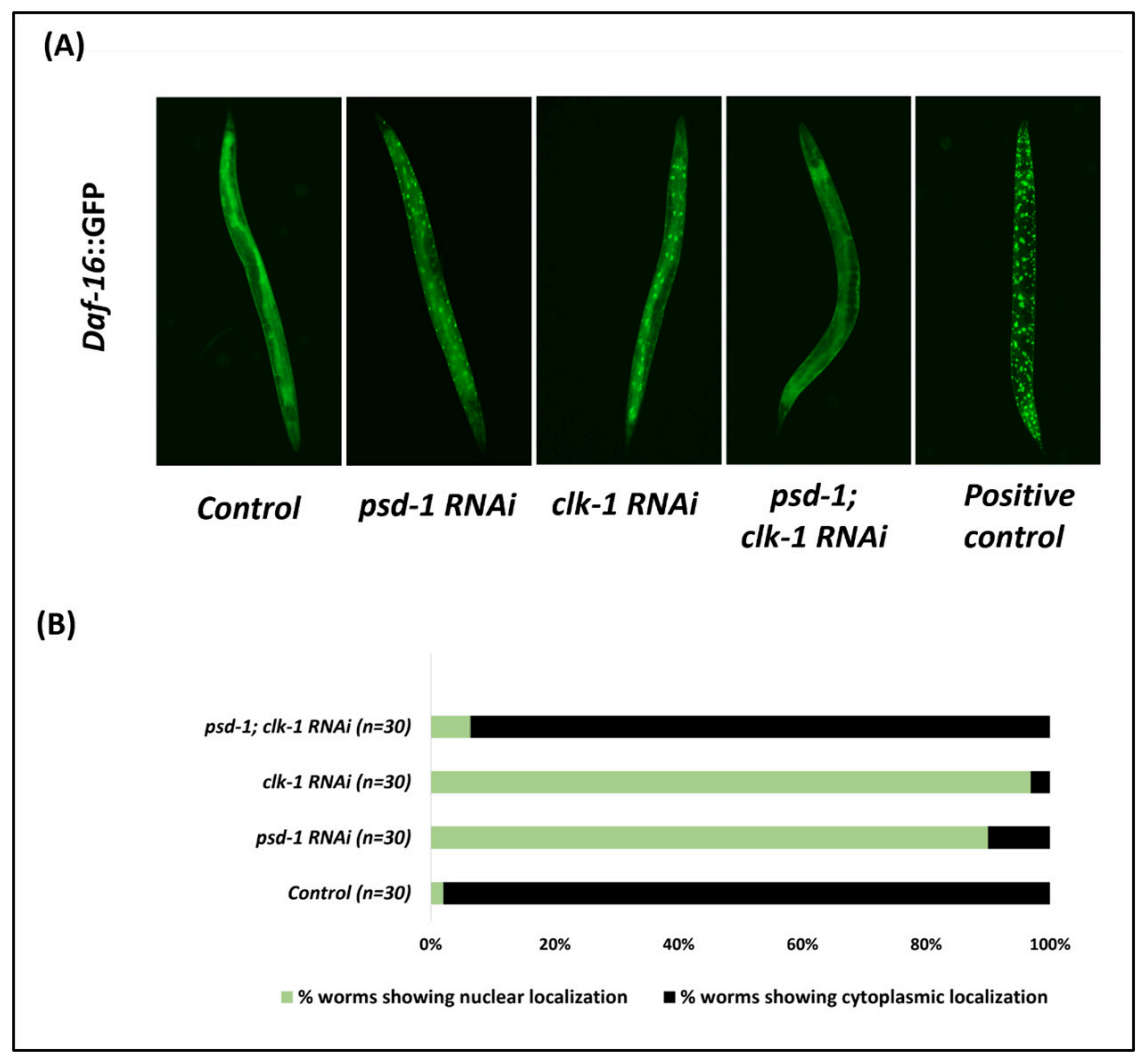
DAF-16 localizes to cytoplasm in double knockdown worms alongside the rescue of physiological phenotypes. (**A**) Representative images of cytoplasmic localization of DAF-16 in control and *psd-1*; *clk-1* double knockdown worms, nuclear localization of DAF-16 in *psd-1* and *clk-1* knockdown worms. Heat-stressed, age-synchronized adult control worms were used as a positive control (*n* = 30 worms per condition). (**B**) Quantification of DAF-16::GFP localization (cytoplasmic or nuclear) is shown as a percentage of total worms scored. Data represent mean ± SD from three independent biological replicates.

To know whether mitochondrial stress signaling pathways were activated in knockdown worms due to increased oxidative stress, we monitored subcellular DAF-16 localization using DAF-16::GFP reporter strain. *psd-1* knockdown worms showed nuclear localization of DAF-16, indicating the intracellular stress response (Figure 6A). These results indicate that loss of PSD-1 changes cellular homeostasis involved in maintaining the mitochondrial redox balance and triggers stress response mediated by DAF-16.

We next asked whether the stress response observed in *psd-1* knockdown worms was changed in double knockdowns, consistent with the rescue of development, lifespan, ROS levels, and respiration. To address this, we tracked DAF-16::GFP localization in *clk-1* RNAi and *psd-1*; *clk-1* double knockdown worms. *clk-1* RNAi worms showed DAF-16 nuclear localization as expected. Surprisingly, double knockdown worms did not show any nuclear localization of DAF-16 as the stress is relieved because of the reduced ROS levels (Figure 6A). Distribution of fluorescence signals was categorized into cytoplasmic and nuclear fractions, and corresponding distribution graphs were shown in Figure 6B.

### 3.7. Synthetic Rescue of Lipid Homeostasis in Double Knockdown Worms

**Figure 7:**
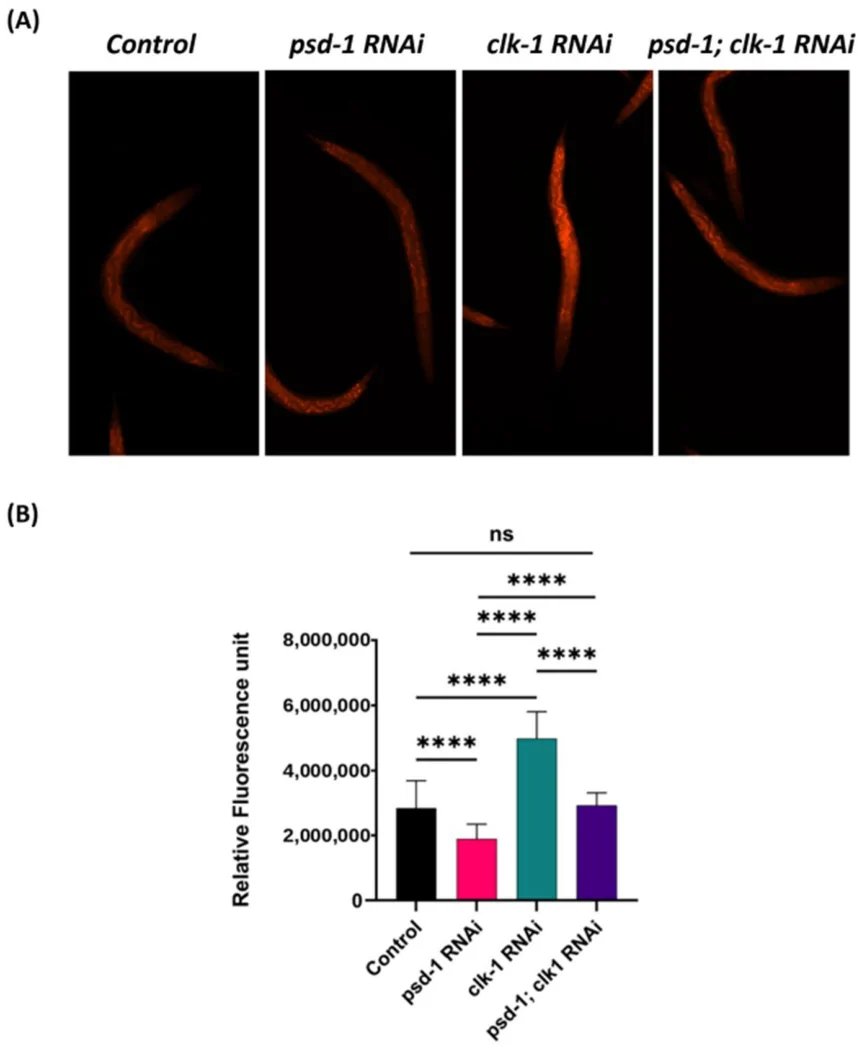
Co-knockdown of *psd-1* and *clk-1* restores lipid droplet content. (**A**) Representative Nile red staining images of control, *psd-1 RNAi*, *clk-1 RNAi* and *psd-1*; *clk-1 RNAi* worms. (**B**) Quantification of Nile red fluorescence intensity (relative to body fat content) in all the four conditions (*n* = 22 worms per condition). Data represent mean ± SD from three biological replicates; statistical significance was determined by Tukey’s multiple comparisons two-way ANOVA (**** *p* < 0.0001, ns = not significant).

To check whether the rescue is correlated with alterations in energy storage, we examined fat storage in knockdown worms using Nile red stain, which selectively stains lipid droplets. We observed a reduction in lipid droplets and overall body fat content in *psd-1* knockdown worms (*p* < 0.0001) when compared to control worms. In contrast, *clk-1* knockdown worms showed an increase in lipid droplet accumulation relative to control (*p* < 0.0001). *psd-1*; *clk-1 RNAi* knockdown worms showed increased lipid droplets relative to *psd-1 RNAi* (*p* < 0.0001), similar to control (control vs. *psd-1*; *clk-1 RNAi*; *p* = 0.9716) (Figure 7A); quantification is shown in Figure 7B. This fat storage profile reveals the rescue of systemic energy homeostasis. These findings suggest that altering mitochondrial ubiquinone biosynthesis triggers a protective rewiring effect by allowing the organism to maintain stable lipid storage reserves through homeostatic adaptation.

To determine whether the developmental and lifespan rescue observed in *psd-1*; *clk-1* double knockdown worms reflect restoration of mitochondrial PE levels or a functional bypass independent of the primary lipid defect, we quantified mitochondrial phospholipids by TLC in double knockdown worms. Mitochondrial PE levels in *psd-1*; *clk-1* double knockdown worms remained reduced when compared to control (*p* = 0.0004), and PS levels remained elevated (*p* = 0.0039) (Supplementary Figure S4) comparable to the *psd-1* single knockdown. This indicates that the phenotypic rescue conferred by *clk-1* co-knockdown occurs without correction of the primary phospholipid deficit, supporting a model in which *clk-1* downregulation relieves downstream ETC-mediated retrograde stress signaling rather than restoring mitochondrial PE homeostasis itself.

## 4. Discussion

In this work, we demonstrated that PSD-1-mediated PE biosynthesis is crucial for maintaining organismal health, development, and longevity in *C. elegans*. Our data showed that *psd-1* deficiency causes developmental delay, reduced lifespan, induced stress response, impaired mitochondrial health, reproductive capacity and lipid homeostasis. These phenotypic characteristics highlight the conserved role of PSD-1 in protecting the integrity of mitochondria across eukaryotes. These features are also closely associated with mitochondrial abnormalities caused by decreased mitochondrial ETC activity and altered cristae morphology. As mentioned in the previous study by Zhao et al., (2019), insufficient PE biosynthesis in mitochondria due to biallelic mutations in human *PISD* gene caused a severe multisystem disorder characterized by skeletal dysplasia and cataracts [11]. Similarly, *PSD1* deletion in yeast led to abnormal mitochondrial morphology, decreased PE levels, stunted growth, impaired respiration and increased ROS levels [13,35]. All these characteristic features are closely linked to mitochondrial defects that are caused by decreased ETC activity in *C. elegans* animal model.

Based on the above observations on phenotypic characterizations, we wanted to know if physiological dysfunctions caused by PSD-1 loss could be modulated genetically. Therefore, we investigated the genetic interaction between *psd-1* and *clk-1*. Combined knockdown of *psd-1* with *clk-1* rescued developmental phenotype (Figure 3A), significantly restored lifespan (Figure 3D) and improved mitochondrial activity (Figure 5A) and lipid metabolism (Figure 7) when compared to *psd-1* single knockdown. Although CLK-1 co-knockdown rescues the phenotypes caused by PSD-1 loss, it cannot compensate for the loss of PE levels in mitochondrial membranes (Supplementary Figure S4). This suggests that the genetic interaction between *psd-1* and *clk-1* represents a functional and bioenergetic rewiring, rather than structural correction caused by phospholipid loss. Such genetic interaction between *psd-1* and ETC component has been previously reported in yeast [14] and to our knowledge this is the first interaction described in a whole animal model.

This genetic link between phospholipid and ubiquinone pathways is consistent with the previous observation where CoQ levels increased following *CHO2* deletion in the yeast [14]. CHO2 is a PE methyltransferase that transfers methyl groups to PE to form PC. Deletion of *CHO2* may indirectly increase PE levels in the mitochondria by accumulation of the substrate of CHO2. These observations may reflect a general tendency of CoQ pools being linked to mitochondrial phospholipid levels.

To evaluate whether *clk-1* co-knockdown functionally rescues mitochondrial dysfunction, we assessed organellar health across four measures: respiration (OCR), electron leakage (ROS), retrograde stress signaling (DAF-16), and metabolic output (Nile red). Concurrent improvement across all four measures supports the conclusion that a broad functional and bioenergetic rescue has been achieved. We hypothesize two non-exclusive mechanisms to explain the phenotypic rescue outcomes of the *psd-1*; *clk-1* double knockdown worms. The first depends on the metabolic rewiring. To understand how *psd-1* loss is sensed and communicated in the organism, we examined the conserved stress-responsive transcription factor DAF-16/FOXO, which is an effector of the insulin/IGF-1 signaling (IIS) pathway in *C. elegans*. Under normal conditions, the insulin-like receptor DAF-2 signals AGE-1/PI3K-AKT-1/2-SGK-1 kinase cascade to phosphorylate DAF-16 so that it remains in the cytoplasm. Under stress conditions, DAF-16 translocates into the nucleus to activate antioxidants and longevity-related genes. In *psd-1* knockdown worms, DAF-16 is localized in the nucleus, which is consistent with the significantly elevated ROS levels and impaired respiration we measured. PE depletion is sufficient to suppress the activity of IIS and activate this stress-responsive pathway.

*clk-1* knockdown worms also showed DAF-16 nuclear localization consistent with previous reports indicating lifespan extension in long-lived mitochondrial mutants occurs through ROS-mediated DAF-16 activation [36]. Mutation in this gene leads to accumulation of the precursor demethoxyubiquinone (DMQ9), which slows down the physiological processes while extending the lifespan [37]. This metabolic slow-down reflects the well documented phenotypes of *clk-1* mutants in *C. elegans* and is consistent with the theory of mitohormesis, a phenomenon in which mild levels of mitochondrial stress trigger protective adaptations [38,39]. We propose that the secondary restriction of ubiquinone biosynthesis by *clk-1* co-knockdown may act as such a mitohormetic trigger, lowering chronic electron leakage without correcting the primary PE loss caused by *psd-1* knockdown. It is also possible that parallel stress-responsive pathways act alongside DAF-16 to establish this balance; in particular, mild restriction of ETC activity by *clk-1* downregulation is a recognized mitohormetic trigger in other systems and could plausibly engage the mitochondrial unfolded protein response (UPR-mt) or baseline autophagy and selective mitophagy [39]. These pathways were not directly measured in the present study, and studying them is an important direction for future work. In double knockdown worms, PE depletion along with reduced levels of CLK-1 may trigger cellular signals that promote mitochondrial quality and organism growth. This facilitates mitochondria to efficiently use oxygen and reduce ROS levels despite the shortage of phospholipid. Reduced ROS levels thereby retain DAF-16 protein in the cytoplasm. This double knockdown rewires the damaged ETC to work efficiently without fixing the structural defect caused by PSD-1 loss.

The second mechanism could be the compensation of lipid metabolism and homeostatic adaptation. Loss of PE might have triggered compensatory lipid metabolic pathways. Our Nile red staining data (Figure 7A,B) showed severe depletion of lipid droplets in *psd-1* knockdown worms. Interestingly, co-knockdown of *clk-1* might have triggered the metabolism to shift towards lipid accumulation. This adaptive rewiring of lipid metabolism in phenotypic rescue is supported and evidenced by the partial restoration of lipid droplets in double knockdowns.

Our findings may offer a useful perspective on human PISD-related conditions specifically, such as skeletal dysplasia, cataracts, and Liberfarb syndrome phenotypes associated with PISD mutations [11,12] which are characterized by oxidative stress, impaired cellular homeostasis, and bioenergetic failure. Current therapeutic approaches for such conditions largely focus on improving or bypassing ETC function with generalized antioxidants or electron shuttles, or on direct phospholipid/substrate replacement, both of which face efficacy, delivery, or bioavailability challenges in mammalian tissues. Our study demonstrates that modulating ubiquinone biosynthesis does not correct the underlying phospholipid defect but can partially rescue the downstream physiological effects. This idea that a downstream signaling response can be the better therapeutic target than the primary defect is supported by a recent finding in mammalian systems [40]. For example, targeting the retrograde signaling pathway arising from mitochondrial defects improved disease outcomes. The bidirectional relationship between mitochondrial lipids and ETC function has been reported in a recent study showing that assembly of respiratory supercomplexes influences adjacent lipid bilayer environment, concentrating ubiquinone and cardiolipin molecules [41]. This study bridges mitochondrial membrane lipid homeostasis with systemic redox biology rather than viewing phospholipid depletion purely as an irreversible structural defect. Our work investigates how localized membrane alterations trigger ETC instability and ROS leakage and suggests how modulating endogenous electron carrier pathways can influence the cellular redox homeostasis and organismal health span in this model, extending beyond the traditional focus on exogenous antioxidant supplementation.

## 5. Conclusions

This study demonstrates the importance of PSD-1 in mitochondrial function as well as organismal development, lifespan and overall health in *C. elegans*. Our application of lipid staining, genetic epistasis study and GFP reporters provides an understanding of how lipid deficiencies manifest at the organismal level. Remarkably, co-knockdown of *clk-1* along with *psd-1* significantly rescues severe physiological and metabolic defects induced by *psd-1* deficiency. This double knockdown successfully normalizes ROS levels, restores oxygen consumption rates and promotes relocalization of DAF-16/FOXO to the cytoplasm.

These findings show that physiological phenotypes associated with phospholipid depletion are not permanent results of structural damage but instead they are driven by ETC-mediated retrograde stress signals and intracellular oxidative crisis. Our investigation also suggests that, in addition to targeting the fundamental metabolic defect, therapeutically modifying the downstream stress-responsive, longevity-related, and CoQ-associated pathways could be beneficial in mitochondrial diseases. However, it is important to acknowledge certain limitations in this study. While RNAi-mediated knockdown is highly effective, it might not perfectly mimic knockout conditions, and residual gene expression could underestimate the severity of complete loss of function. This difference may be relevant for *clk-1*, which shows developmental delay in knockout worms but did not show any difference in our study. This may reflect a previously reported maternal rescue phenotype of *clk-1* mutants [42]. RNAi-mediated *clk-1* knockdown does not eliminate maternal contribution as cleanly as genetic null mutants and keeps some residual function of *clk-1* intact. CoQ and DMQ9 levels were assigned based on their retention times relative to the CoQ10 standard. Even though this assignment aligns with literature, future work using LC-MS/MS validation needs to be done to confirm the peak profiles. Future research using knockout models could further clarify the precise mechanisms underlying this rescue and the specific contribution of ubiquinone modulation. Future work should also examine which specific phospholipid and lipid-droplet species are altered due to *clk-1* knockdown, and evaluate whether pharmacological ubiquinone modulation could recapitulate the genetic rescue as a potential therapeutic approach for PISD-related patients.

## Data Availability

Data is contained within the article and Supplementary Material.

## Supplementary Materials

The following supporting information can be downloaded at: https://www.mdpi.com/article/10.3390/antiox15091212/s1, Figure S1: Genetic interaction analysis of *psd-1* with genes involved in mitochondrial function, oxidative stress and lipid metabolism during development of *C. elegans.* Representative brightfield images of *C. elegans* subjected to single knockdown individually and double knockdown in combination with *psd-1.* Worms were grown on RNAi feeding plates of different genes and 20 worms were imaged at every 12 h (0, 12, 24, 36 and 48) of development. The red box indicates developmental delay. This experiment was conducted in three independent biological replicates. Scale bar = 300 µm; Figure S2: HPLC chromatogram of CoQ10 reference standard. (A) Representative chromatograms of few CoQ10 standards (2, 5, 10, and 20 µg/mL) detected at 275 nm (bandwidth 4 nm); dashed line indicates CoQ10 retention time (~14.5 min). (B) Calibration curve constructed from peak area vs concentration, showing linear regression equation and coefficient of determination (R^2^). Experiment was conducted in three independent biological replicates; Figure S3: Measurement of intracellular ROS levels. (A) ROS levels in all the four conditions control, *psd-1 RNAi*, *clk-1 RNAi* and *psd-1*; *clk-1 RNAi* worms were measure and quantified using DCFH-DA. *psd-1* RNAi worms showed elevated levels of ROS compared to control worms. Double knockdown worms showed reduced ROS compared to *psd-1* knockdown worms. (B) ROS levels in *clk-1 RNAi* worms compared to control showed significantly increased levels of ROS compared to control worms from 9th hour. Experiment was conducted in three independent biological replicates. Data are presented as mean ± SD (** *p* < 0.01, *** *p* < 0.001 and **** *p* < 0.0001); Figure S4: TLC of mitochondrial phospholipids. (A) Thin-layer chromatography of mitochondrial phospholipids extracted from control, *psd-1* RNAi, *clk-1* RNAi, and *psd-1*; *clk-1* RNAi worms (200 µg mitochondrial protein per condition was used to extract phospholipids), alongside a PE standard. (B) Densitometric quantification of PE band intensity, normalized to control; PE levels did not differ significantly between *psd-1* RNAi and *psd-1*; *clk-1* RNAi, indicating that *clk-1* co-knockdown does not restore mitochondrial PE. (C) Densitometric quantification of PS band intensity, normalized to control, showing significant PS accumulation in *psd-1* RNAi and *psd-1*; *clk-1* RNAi worms. Data are presented as mean ± SD from two technical replicates (** *p* < 0.01, *** *p* < 0.001, ns = not significant; one-way ANOVA with Tukey’s post-hoc test); Table S1: List of qRT PCR primers used in this study.
