# Supplementary material for: Modulating Ubiquinone Biosynthesis Rescues Mitochondrial Dysfunction by Genetic Interaction in a Phosphatidylethanolamine (PE)-Deficient Disease Model
Source: Antioxidants (Basel). 2026 Sep 20;15(9):1212. doi: 10.3390/antiox15091212 (PMC13603120; doi:10.3390/antiox15091212)
Supplement: Supplementary file 1 [file antioxidants-15-01212-s001.zip › antioxidants-4524386-supplementary.pdf]

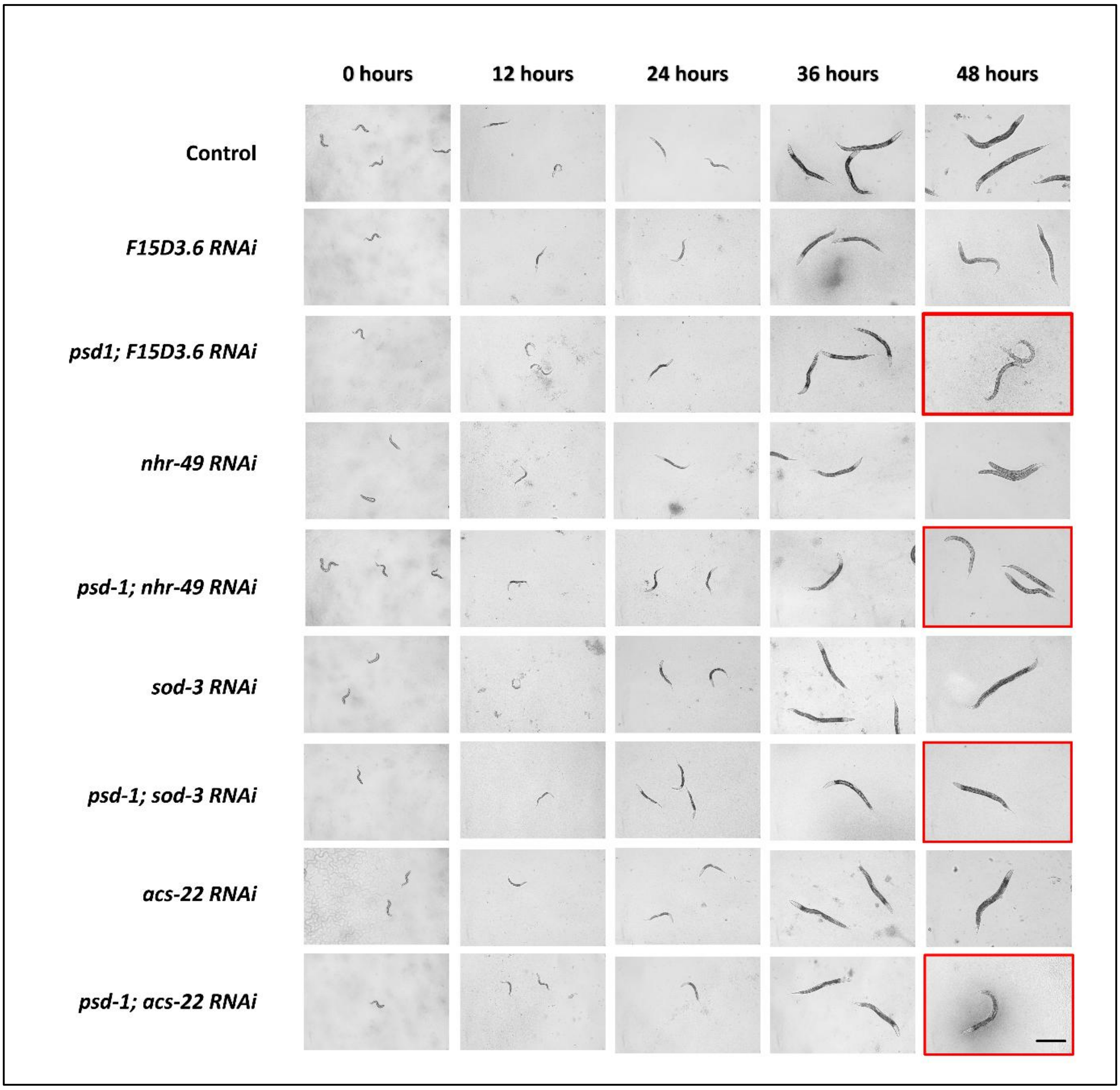

Supplementary Figure S1. Genetic interaction analysis of *psd-1* with genes involved in mitochondrial function, oxidative stress and lipid metabolism during development of *C. elegans.*

Representative brightfield images of *C. elegans* subjected to single knockdown individually and double knockdown in combination with *psd-1.* Worms were grown on RNAi feeding plates of different genes and 20 worms were imaged at every 12 hours (0,12,24,36 and 48) of development. The red box indicates developmental delay. This experiment was conducted in three independent biological replicates. Scale bar = 300 µm.

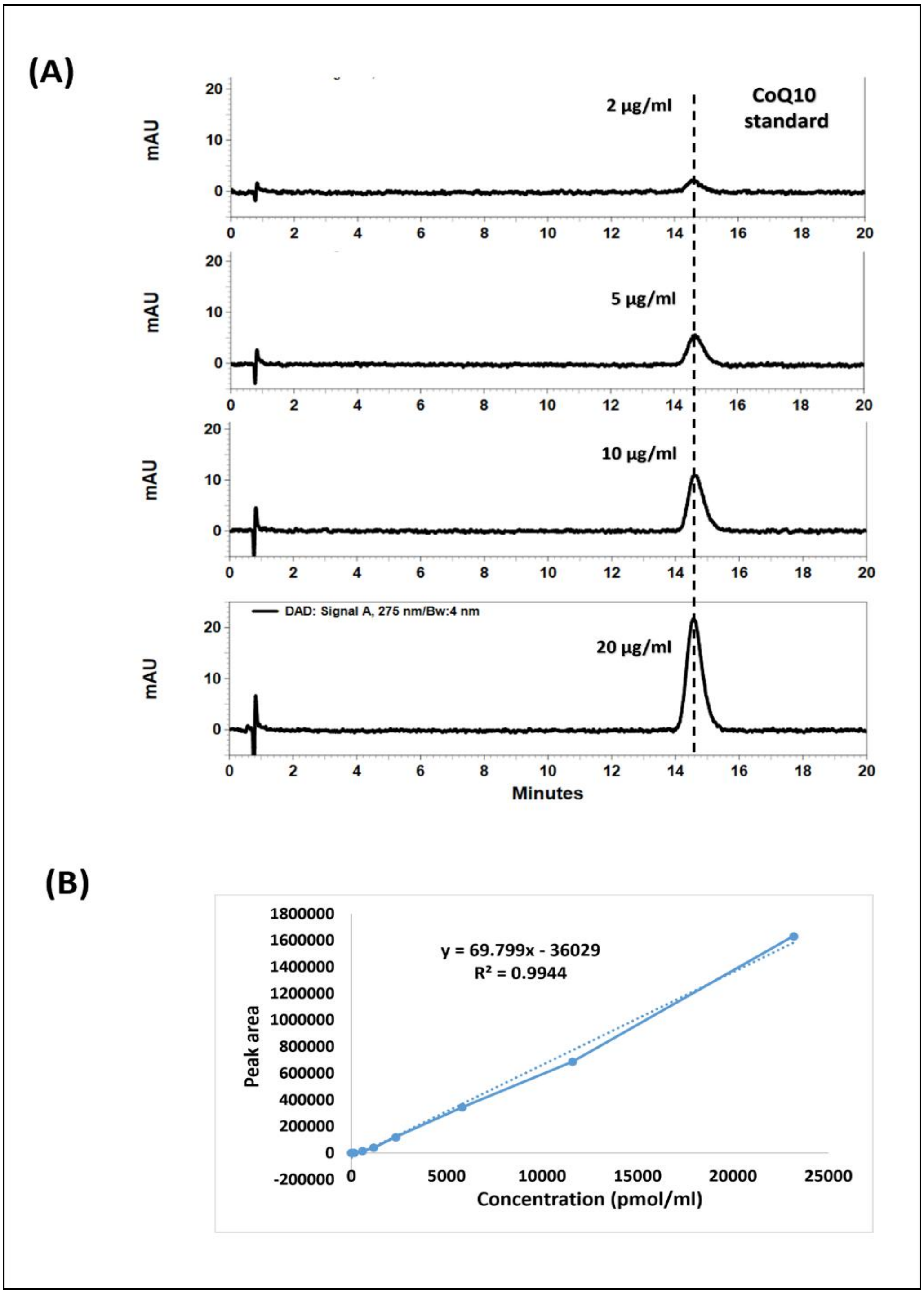

Supplementary Figure S2. HPLC chromatogram of CoQ10 reference standard

(A) Representative chromatograms of few CoQ10 standards (2, 5, 10, and 20 µg/ml) detected at 275 nm (bandwidth 4 nm); dashed line indicates CoQ10 retention time (~14.5 min). (B) Calibration curve constructed from peak area vs concentration, showing linear regression equation and coefficient of determination (R^2). Experiment was conducted in three independent biological replicates.

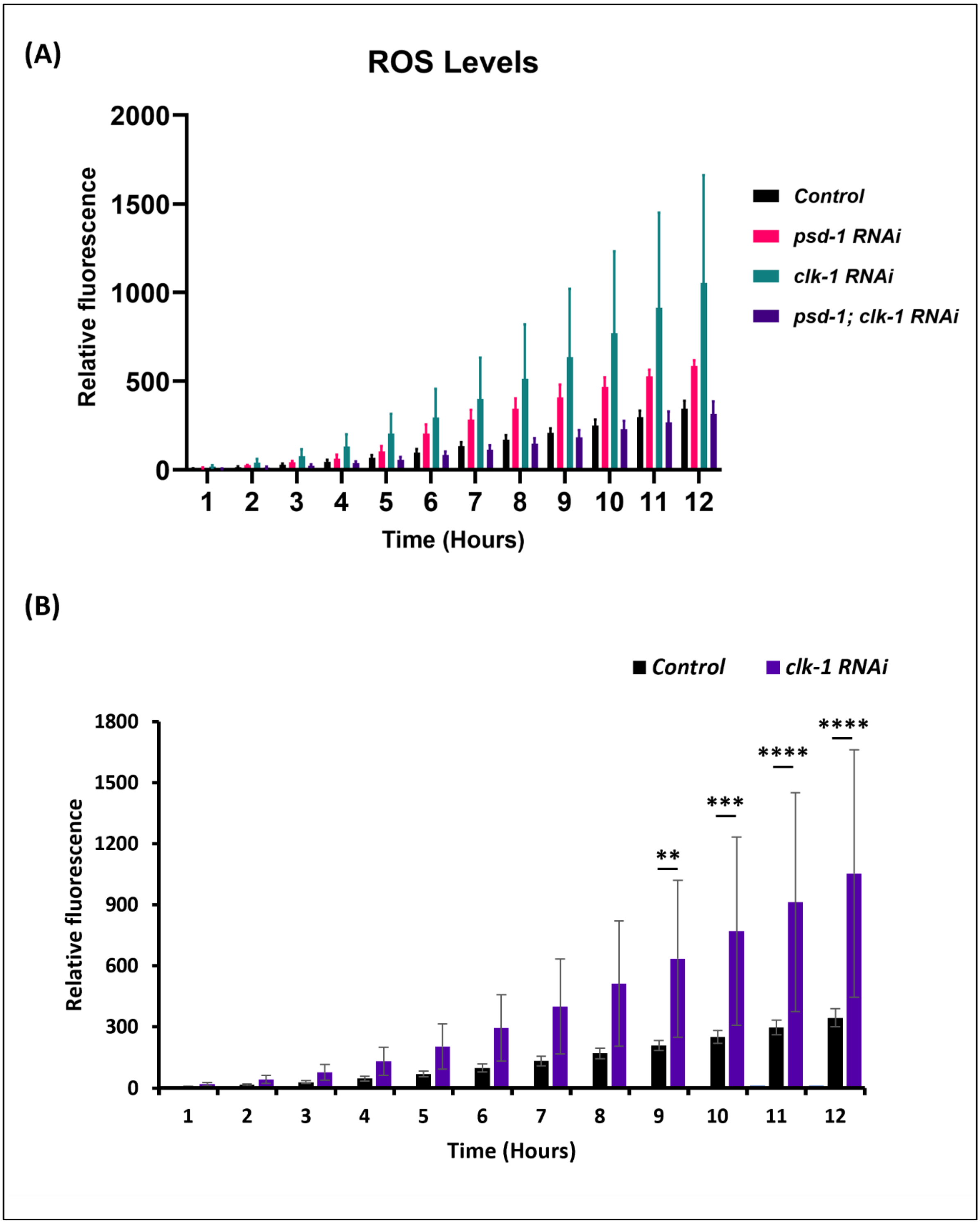

Supplementary Figure S3. Measurement of intracellular ROS levels

(A) ROS levels in all the four conditions control, *psd-1 RNAi, clk-1 RNAi and psd-1; clk-1 RNAi* worms were measure and quantified using DCFH-DA. *psd-1* RNAi worms showed elevated levels of ROS compared to control worms. Double knockdown worms showed reduced ROS compared to *psd-1* knockdown worms. (B) ROS levels in c*lk-1 RNAi* worms compared to control showed significantly increased levels of ROS compared to control worms

from 9th hour. Experiment was conducted in three independent biological replicates. Data are presented as mean ± SD (**p < 0.01, ***p < 0.001 and ****p < 0.0001).

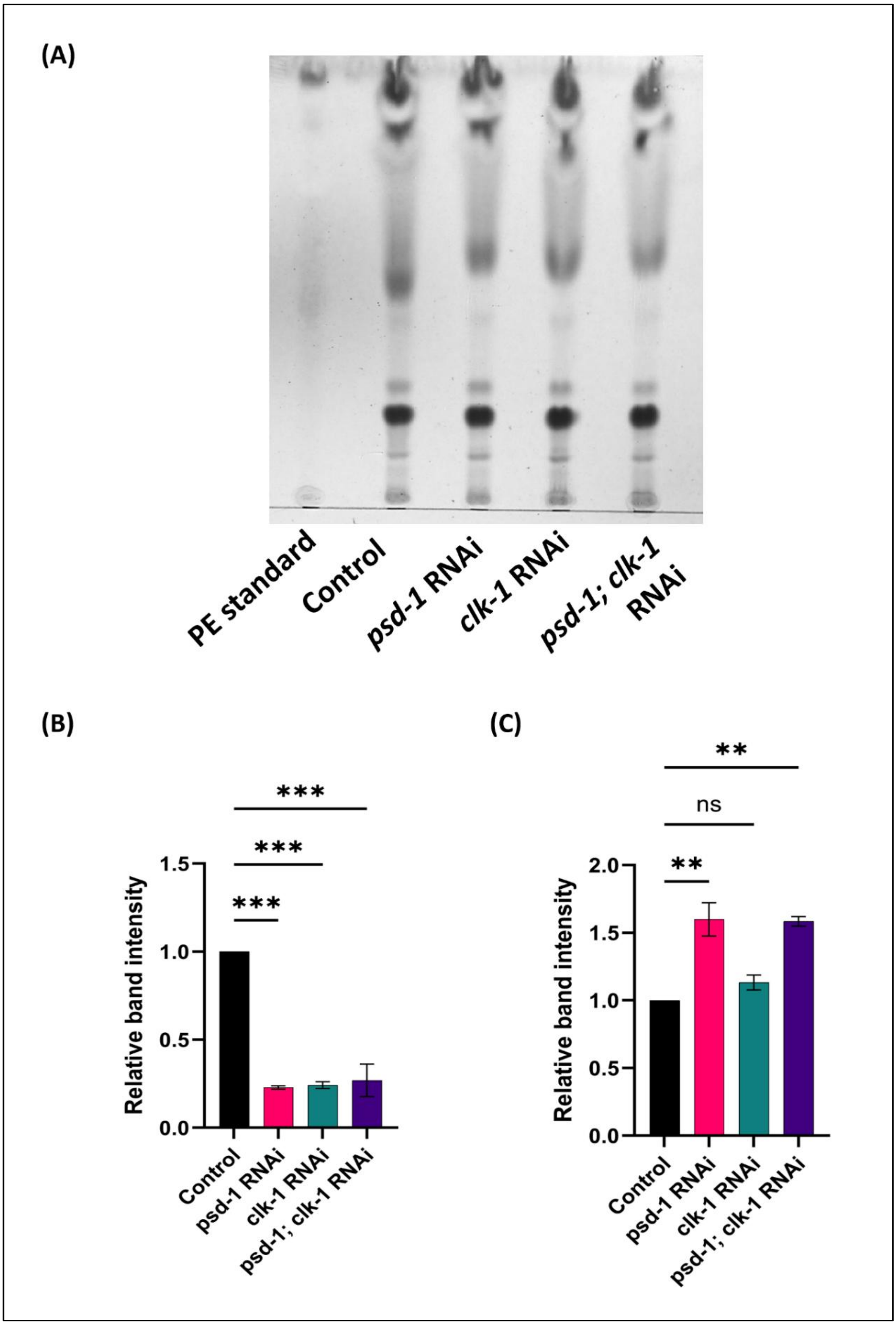

Supplementary Figure S4. TLC of mitochondrial phospholipids.

(A) Thin-layer chromatography of mitochondrial phospholipids extracted from control*, psd-1* RNAi*, clk-1* RNAi, and *psd-1; clk-1* RNAi worms (200 µg mitochondrial protein per condition was used to extract phospholipids), alongside a PE standard. (B) Densitometric quantification of PE band intensity, normalized to control; PE levels did not differ significantly between *psd-1* RNAi and *psd-1; clk-1* RNAi, indicating that *clk-1* co-knockdown does not restore mitochondrial PE. (C) Densitometric quantification of PS band intensity, normalized to control, showing significant PS accumulation in *psd-1* RNAi and *psd-1; clk-1* RNAi worms. Data are presented as mean ± SD from two technical replicates (**p < 0.01, ***p < 0.001, ns = not significant; one-way ANOVA with Tukey's post-hoc test).

Table S1: List of qRT PCR primers used in this study.

**Genes**	**Forward**	**Reverse**
psd-1	CAGTAATTGGAAGATTCGTGTC	CACGTTCACCCGAAACATA
clk-1	CGAGTTGATCATGCTGGAG	CTCAGCAATCGCAATAGCT
act-1	GGAGTCATGGTCGGTATGG	CTTGAGGGTAAGGATACCTCTC
